# New Insights Into Cinnamoyl Esterase Activity of *Oenococcus oeni*

**DOI:** 10.3389/fmicb.2019.02597

**Published:** 2019-11-08

**Authors:** Ingrid Collombel, Chrats Melkonian, Douwe Molenaar, Francisco M. Campos, Tim Hogg

**Affiliations:** ^1^Escola Superior de Biotecnologia, Centro de Biotecnologia e Química Fina, Universidade Católica Portuguesa, Porto, Portugal; ^2^Systems Biology LAB, Vrije Universiteit Amsterdam, Amsterdam, Netherlands; ^3^Plataforma de Inovação da Vinha e do Vinho, Universidade de Trás-os-Montes e Alto Douro, Vila Real, Portugal

**Keywords:** *Oenococcus oeni*, tartrate esters, hydroxycinnamic acids, cinnamoyl esterase, wine

## Abstract

Some strains of *Oenococcus oeni* possess cinnamoyl esterase activity that can be relevant in the malolactic stage of wine production liberating hydroxycinnamic acids that are precursors of volatile phenols responsible for sensory faults. The objective of this study was to better understand the basis of the differential activity between strains. After initial screening, five commercial strains of *O. oeni* were selected, three were found to exhibit cinnamoyl esterase activity (CE+) and two not (CE−). Although the use of functional annotation of genes revealed genotypic variations between the strains, no specific genes common only to the three CE+ strains could explain the different activities. Pasteurized wine was used as a natural source of tartrate esters in growth and metabolism experiments conducted in MRS medium, whilst commercial *trans*-caftaric acid was used as substrate for enzyme assays. Detoxification did not seem to be the main biological mechanism involved in the activity since unlike its phenolic cleavage products and their immediate metabolites (*trans-*caffeic acid and 4-ethylcatechol), *trans*-caftaric acid was not toxic toward *O. oeni*. In the case of the two CE+ strains Oenos^TM^ and CiNe^TM^, wine-exposed samples showed a more rapid degradation of *trans*-caftaric acid than the unexposed ones. The CE activity was present in all cell-free extracts of both wine-exposed and unexposed strains, except in the cell-free extracts of the CE− strain CH11^TM^. This activity may be constitutive rather than induced by exposure to tartrate esters. *Trans*-caftaric acid was totally cleaved to *trans*-caffeic acid by cell-free extracts of the three CE+ strains, whilst cell-free extracts of the CE− strain CH16^TM^ showed significantly lower activity, although higher for the strains in experiments with no prior wine exposure. The EstB28 esterase gene, found in the genomes of the 5 strains, did not reveal any difference on the upstream regulation and transport functionality between the strains. This study highlights the complexity of the basis of this activity in wine related *O. oeni* population. Variable cinnamoyl esterases or/and membrane transport activities in the *O. oeni* strains analyzed and a possible implication of wine molecules could explain this phenomenon.

## Introduction

Hydroxycinnamic acids (HCAs) are a group of phenolic acids and are abundantly present across the plant kingdom. These compounds have many roles in many aspects of biology – both in the plants in which they are produced and in bacteria, fungi, plants and animals that interact with them. Examples of these include post-ingestion effects, through food and beverage consumption, on animals and humans (Boudet, 2007; Prasad et al., 2011; Shahidi and Ambigaipalan, 2015; Calabriso et al., 2016) and plant–fungi signaling through soil diffusion (Ragonezi et al., 2014).

In grapes, HCAs are mostly encountered in forms that are covalently bound to tartaric acid, glucose and the ethyl group, in the vacuoles of the skin and pulp cells. In wines, free HCAs may act as color stabilizers, antimicrobial agents and flavor precursors (Campos et al., 2003; Hernández et al., 2006; Bouzanquet et al., 2012; Lima et al., 2018).

**FIGURE 1:**
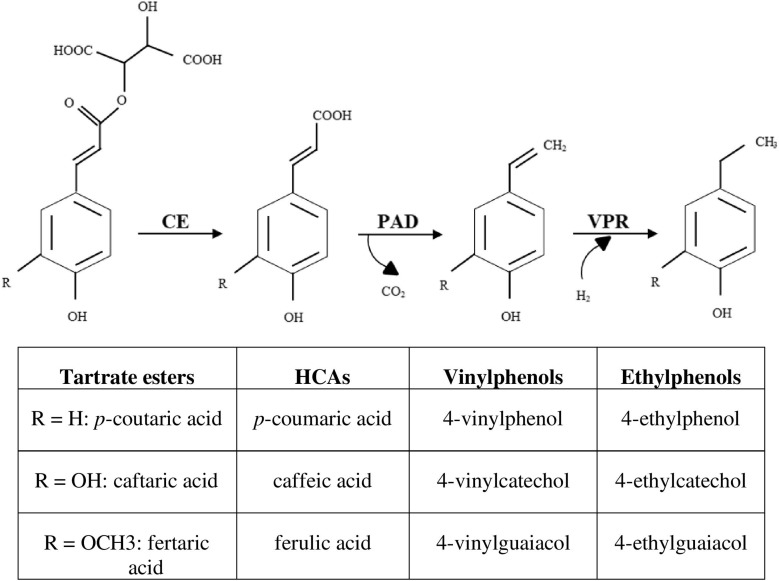
Enzymatical activities linking the production of volatile phenols from its tartrate ester forms. CE, cinnamoyl esterase; PAD, phenolic acid decarboxylase; VPR, vinylphenol reductase; HCAs, hydroxycinnamic acids.

Caffeic, *p*-coumaric and ferulic acids are HCAs and substrates for enzyme systems of the wine spoilage yeast *Brettanomyces/Dekkera bruxellensis* and a number of wine lactic acid bacteria (LAB), through which volatile phenols are produced and can be responsible for sensory faults in wines (Couto et al., 2006; Kheir et al., 2013; Cappello et al., 2016). The most commonly reported pathway for the microbial metabolization of HCAs involves two enzyme systems: a phenolic acid decarboxylase (PAD) and a vinylphenol reductase (VPR) (Figure 1). PAD and VPR activities are variable among the strains and principally modulated by the nature and concentration of the substrate (Barthelmebs et al., 2000; Silva et al., 2011; Filannino et al., 2015; Rosimin and Kim, 2015; Sturm et al., 2015; Cappello et al., 2016). Some *B. bruxellensis* strains have been observed to directly produce ethylphenols from *p*-coumaroyl glucose, feruloyl glucose and ethyl coumarate, but not from *p*-coumaroyl and feruloyl L-tartrate esters (coutaric and fertaric acids) (Hixson et al., 2012, 2016).

Malolactic fermentation (MLF) is a desirable step in the vinification process of most red wines and can be spontaneous, due to grape/winery LAB or induced by selected starters chosen according to the type and quality of wine desired. Due to its tolerance to alcohol content and acidity, *Oenococcus oeni* strains are usually the most predominant LAB that perform the MLF. According to Ribéreau-Gayon et al. (2006), the HCAs that are precursors of volatile phenols are present in wine mainly as their tartrate esters, with *trans*-caftaric acid, the tartaric ester of *trans*-caffeic acid, being the most abundant. These molecules have not yet been described as substrates for the PAD enzymes and so are apparently not direct precursors of volatile phenols. During MLF, the release of free HCAs has been previously linked to the disappearance of its corresponding tartrate ester form (Hernández et al., 2006; Cabrita et al., 2008), possibly linked to the activity of some native grape microorganisms (Zepeda-Mendoza et al., 2018).

Cinnamoyl esterases (CE), also called feruloyl esterases, ferulic acid esterases or hydroxycinnamoyl esterases, have been studied in many microorganisms (*Lactobacillus*, *Bacillus*, *Bifidobacteria*) and commercial enzyme preparations used in winemaking, and are described as enzymes involved in the release of HCAs from their esterified forms (Donaghy et al., 1998; Comino et al., 2014; Fia et al., 2014; Fritsch et al., 2017). Cinnamoyl esterase activity is apparently inducible and depends both on the substrates present and on the specific strain (Brezillon et al., 1996). Crepin et al. (2004) classified the microbial CE into four groups based on substrate preferences and supported by primary sequence identity. Ethyl ferulate, methyl ferulate, methyl caffeate, methyl *p*-coumarate, methyl sinapinate, and chlorogenic acid are the most common esters cleaved by the CE studied (Crepin et al., 2004). Some CE were reported to be extracellular (Brezillon et al., 1996), whilst others were found to be located within the cell (Gobbetti et al., 1996; Castillo et al., 1999). A few of these enzymes produced during fermentation have yet been purified from various bacteria (*Lactobacillus acidophilus*, *Lactobacillus johnsonii*, *Lactobacillus plantarum*, *Lactobacillus helveticus*) and fungi (*Penicillium pinophilum*, *Aspergillus awamori*) and characterized to some extent (Castanares et al., 1992; Wang et al., 2004; Kanauchi et al., 2008; Lai et al., 2009; Esteban-Torres et al., 2013, 2015; Song and Baik, 2017). However, none of the microorganisms producing these activities studied are wine-related nor are the cinnamate esters of tartaric acid previously tested as substrates.

Lactic acid bacteria possess a substantial collection of enzymes involved in the synthesis and hydrolysis of esters (Sumby et al., 2010) including the wine-associated LAB *O. oeni* and *L. hilgardii*, catabolizing volatiles aromatic esters and of which the intracellular esterases have been characterized by Sumby et al. (2009, 2013). Among LAB, only *O. oeni* Oenos^TM^ and CiNe^TM^ strains in wine and the probiotic intestinal bacterium *Lactobacillus johnsonii* NCC 533 have been found to possess the cinnamoyl esterase activity enable to cleave tartaric salts from phenolic acids (Figure 1) (Bel-Rhlid et al., 2012; Burns and Osborne, 2013; Chescheir et al., 2015; Madsen et al., 2016; Zepeda-Mendoza et al., 2018). Substrate for this enzymatic activity is limited to *trans*-isomers of the tartrate esters (Hernandez et al., 2007).

Although the production of volatile phenols from phenolic acids has been widely studied in wine, much less is known about prior processes that determine the availability of free precursor molecules from tartrate esters. The objective of this current study is to better understand the differences between LAB strains regarding their cinnamoyl esterase activity.

## Materials and Methods

### Microbial Cultures

#### Source and Preparation of Cultures

Seven commercial *O. oeni* strains were used in this study; Viniflora^®^ Oenos^TM^, CH11^TM^, CH16^TM^, CH35^TM^, CiNe^TM^ from Ch. Hansen (Hørsholm, Denmark) as well as Enoferm Alpha^TM^ and Lalvin VP41^TM^ from Lallemand (Montreal, Canada). The wine-related strain *L. plantarum* NOVA^TM^ from Ch. Hansen and the non-wine related strains *L. plantarum* NCFB 1752, *Pediococcus damnosus* NCFB 1832T, *P. pentosaceus* NCFB 990T and *L. brevis subsp. gravesensis* NCFB 1749T from the National Collection of Food Bacteria (Reading, UK) were also tested for their cinnamoyl esterase activity. Three Port-wine isolates were also screened: *L. hilgardii* ESB 19, *L. fructivorans* ESB 92 and *L. collinoides* ESB 99, isolated by Couto and Hogg (1994), from the Escola Superior de Biotecnologia, Portuguese Catholic University (Porto, Portugal). Pre-cultures were grown aerobically at 25°C with no agitation to late exponential phase (Absorbance of about 1.6 AU at 600 nm for *Lactobacillus* and *Pediococcu*s strains and about 0.9 AU for *Oenococcus* strains except for CiNe^TM^ about 0.6 AU and CH35^TM^ about 0.4 AU) in liquid MRS (de Man, Rogosa and Sharpe) medium from BIOKAR Diagnostics (Allonne, France) supplemented with 10 mg/L cycloheximide (Sigma-Aldrich, Steinheim, Germany) and 5% (v/v) absolute ethanol (Carlo Erba, Val de Reuil, France) since ethanol added at this concentration has previously been found to stimulate growth of many wine LAB strains (Couto and Hogg, 1994). The initial pH of the liquid MRS medium was adjusted to 4.5 using a (12 M) hydrochloric acid solution. Absorbance was measured (at 600 nm wavelength) with an UV/VIS UNICAM 8620 spectrophotometer (UNICAM, Cambridge, United Kingdom) and optical cells of 1 cm path length.

#### Toxicity Evaluation of *Trans*-Caftaric Acid, *Trans*-Caffeic Acid and 4-ehylcatechol Against Wine *O. oeni*

In order to study whether the cinnamoyl esterase activity in *O. oeni* is somehow related to a detoxification effect, the toxic levels of *trans-*caftaric acid and its immediate phenolic metabolites (*trans-*caffeic acid and 4-ethylcatechol) were evaluated in MRS broth supplemented with ethanol and cycloheximide (at 5% v/v and 10 mg/L, respectively) and with pH adjusted to 4.5.

Solutions of 4-ethylcatechol (4EC – purity 95%), t*rans*-caftaric and *trans*-caffeic acids (Sigma-Aldrich, Germany) were prepared immediately before use from the pure compounds by dissolving the appropriate amounts in ethanol (99.5% v/v). Fresh cultures of the five *O. oeni* strains from Ch. Hansen were cultivated in 96-well × 300 μL microplates containing MRS broth supplemented with 300 mg/L and 150 mg/L of *trans*-caftaric acid, 170 mg/L and 85 mg/L of *trans*-caffeic acid and 130 mg/L and 65 mg/L of 4EC, representing equivalent molar concentrations (1 and 0.5 mmol/L respectively) for all compounds. Cultures were grown at 25°C with no agitation for 9 days with 1 day as interval of absorbance measurement. Absorbance was measured (at 600 nm wavelength) with a Synergy^TM^ HTX Multi-Mode Microplate Reader from BioTek Instruments (Winooski, VT, United States).

### Enzymatic Activity

#### Wine as a Natural Source of Tartrate Esters

A red wine (13.28% (v/v) alcohol, pH 3.79, 1.00 g/L malic acid and 1.35 g/L lactic acid) from the Touriga Franca variety collected in the Douro region (in Northern Portugal) from the 2017 harvest (stored at 4°C before use) was pasteurized for 3 min at 50°C to reduce competing microbiota whilst minimizing the changes in relevant metabolite composition. Prior wine contamination was checked by the drop-count technique (Miles et al., 1938) on MRS agar medium, prepared with the same composition as liquid MRS medium with 2% (w/w) agar from Liofilchem (Roseto degli Abruzzi, Italy). Plates were incubated at 25°C for 8–10 days before LAB counting (detection limit 500 CFU/mL).

In most of the following experiments, bacteria were cultivated in liquid MRS medium with 30% pasteurized wine [wine-exposed: pH 4.29, 7.48% (v/v) alcohol]. The HCAs/tartrate derivatives composition of the preparations is shown in Table 1.

**TABLE 1:** Concentrations in mg/L of HCAs and their tartrate esters in a mix of 30% pasteurized red wine and 70% MRS broth.

*Trans*-caftaric acid	52.76 ± 0.30
*Trans*-caffeic acid	0.56 ± 0.04
*Trans*-coutaric acid	23.79 ± 0.29
*Trans-p-*coumaric acid	1.58 ± 0.15
*Trans*-fertaric acid	2.34 ± 0.05
*Trans-*ferulic acid	0.02 ± 0.01

*Values represent the mean of three values ± standard deviation.*

#### Cinnamoyl Esterase Activity Screening

Pre-cultures of LAB were transferred to 100 mL flasks containing 30 mL pasteurized wine and 70 mL MRS broth (30% pasteurized wine). The HCAs/tartrate esters composition of the preparations is given in Table 1.

After growing at 25°C with no agitation and reaching a concentration of ∼ 9.26 (±0.21) log CFU mL^–1^ in 2–4 days for *Lactobacillus* and *Pediococcu*s strains and 5–8 days for *Oenococcus* strains, 1 mL samples were collected and stored at −20°C before High-Performance Liquid Chromatography (HPLC) analysis. Lactic acid bacteria counts were done in triplicate using the drop-count technique (Miles et al., 1938). Each individual assay was performed in duplicate.

#### Location of Cinnamoyl Esterase Activity

Cultures of the five *O. oeni* strains from Ch. Hansen were prepared in sterile flasks containing 250 mL liquid MRS medium (unexposed: pH 4.5, 5% (v/v) alcohol) and 175 mL liquid MRS medium mixed with 75 mL (30%) of pasteurized wine (wine-exposed: pH 4.29, 7.48% (v/v) alcohol) and incubated at 25°C with no agitation until the stationary growth phase (5–7 days depending on the strain). After reaching an absorbance (at 600 nm) of 0.6 AU (10^8^ to 10^9^ CFU mL^–1^), cells were harvested by centrifugation (7500 g for 10 min at 4°C). The absorbance was measured for unexposed samples by spectrophotometry (UNICAM, Cambridge, United Kingdom). Bacterial counts were done in triplicate for both unexposed and wine-exposed samples using the drop-count technique (Miles et al., 1938).

Among the 13 lysis protocols tested on the CE+ *O. oeni* strain Oenos^TM^, only two (one enzymatic and one mechanical) were able to satisfactorily disrupt the membrane and therefore were used for the following experiment. The supernatants of the three CE+ strains (extracellular parts) were collected and the pellet was washed twice with sodium phosphate buffer (50 mM, pH 7) for the enzymatical protocol and NaCl 0.15 M for the mechanical protocol. For the enzymatical protocol, the cells were resuspended in 6 mL sodium phosphate buffer (50 mM, pH 7) with 10 mg/mL of lysozyme from Thermo Fisher Scientific (Waltham, MA, United States), split in four 2 mL sterile tubes containing 200 μL autoclaved glass beads 1 mm diameter (Sigma Aldrich, Germany) and incubated for 1 h at 37°C. Bacterial cells were then disintegrated three times 20-s cycles with FastPrep^®^-24 Classic Instrument (MP Biomedicals, Santa Ana, CA, United States) set with a speed of 4 m/s, and cooled 5 min in ice in between each beads-beating. For the mechanical protocol, the cells were resuspended in 6 mL PBS (pH 7.4) and the protein extract was obtained by sonication using a Bandelin Sonopuls HD 2200 homogenizer fitted with an UW 200 probe (Bandelin Electronics, Berlin, Germany) for a total of 20 min. Each disruption cycle lasted 3 min (2 min for the last one) with the power set to 20%. During sonication and 3 min cooling in between each disruption cycle, the cell suspension was immersed in ice. The probe was washed with commercial bleach and ethanol 96.0% v/v before changing of sample. A control without bacteria was also performed for each cell-lysis protocol.

The suspension of disintegrated cells was centrifuged (15000 *g* for 20 min at 4°C) to sediment the disrupted cell fraction (cell debris). The cell debris were washed twice with 5 mL of NaCl 0.15 M and resuspended in 5 mL of sterile, cold distilled water. The efficiency of the lysis protocols was evaluated using the drop-count technique on the cell suspension before lysis and on the cell debris after washing and resuspension.

The extracellular part and the cell extract of each culture were filtered using sterile filters of 0.22 μm pore size from Elkay Laboratory Products (Basingstoke, United Kingdom). *Trans*-caftaric acid (only available tartrate ester in the market), previously dissolved in ethanol (99.5% v/v), was added at a concentration of 10 mg/L and the mixture was incubated at 30°C for 16 h. Samples were collected before and immediately after substrate addition and after incubation. Samples were analyzed to test their cinnamoyl esterase activity by HPLC analysis.

#### Suitability of Ferulic Acid Methyl Ester as Substrate for the Cinnamoyl Esterase Activity

A parallel experiment was conducted in liquid MRS with and without 100 mg/L of ferulic acid methyl ester, previously dissolved in ethanol- that has been used as substrate for the cinnamoyl esterase activity in other studies of this type (Crepin et al., 2003; Esteban-Torres et al., 2013, 2015). The esterase activity was evaluated in the extracellular part and the cell extract of the five *O. oeni* strains by adding 100 mg/L of ferulic acid methyl ester before incubation for 16 h at 30°C.

#### Measurement of the Cinnamoyl Esterase Activity

The three CE+ *O. oeni* strains Oenos^TM^, CiNe^TM^ and CH35^TM^ were grown in pure MRS liquid medium (unexposed) or in 70% MRS liquid medium with 30% pasteurized wine (wine-exposed) as described in the previous sections. The cells were harvested by centrifugation (7500 *g* for 10 min at 4°C), and the pellets were washed twice with KH_2_PO_4_ buffer (0.15 M, pH 4.5, 9% EtOH), as described by Campos et al. (2003) with the cells being resuspended in the same buffer with 10 mg/L of *trans*-caftaric acid. The cinnamoyl esterase activity was measured by following the increase in concentration of free *trans*-caffeic acid over 5 h in 25°C by HPLC.

In order to evaluate the possible stimulation of the cinnamoyl esterase activity of the CE + strain Oenos^TM^, cultures were grown without agitation for 5 days and 10 h at 25°C in: 1 – pure liquid MRS medium (unexposed: pH 4.5, 5% (v/v) alcohol), 2 – 10% pasteurized wine (pH 4.43, 5.83% (v/v) alcohol), 3 – 20% pasteurized wine (pH 4.36, 6.66% (v/v) alcohol), 4 – 30% pasteurized wine (pH 4.29, 7.48% (v/v) alcohol, around 50 mg/L of natural *trans-*caftaric acid as shown in Table 1), 5 – 50 mg/L of *trans*-caftaric acid (pH 4.5, 6.7% (v/v) alcohol), 6 – pure liquid MRS medium incubated first for 5 days with substitution by 20% pasteurized wine and incubated a second time for 10 h and 7 – pure liquid MRS medium incubated first for 5 days followed by an addition of 50 mg/L *trans*-caftaric acid and incubated a second time for 10 h. The cells were harvested by centrifugation and the pellets were washed with KH_2_PO_4_ buffer (0.15 M, pH 4.5, 9% EtOH) and resuspended in the same buffer with 10 mg/L of *trans-*caftaric acid. The cinnamoyl esterase was measured over 13 h in 25°C by HPLC.

#### Bioinformatics Analysis

The entire genomes of the five commercial *O. oeni* strains were provided by Ch. Hansen and some were available in the GenBank sequence database [GenBank accession numbers: CiNe^TM^ AZJV00000000 (Dimopoulou et al., 2014); CH35^TM^ ALAG00000000 (Borneman et al., 2012)].

Firstly, to predict the open reading frames (ORFs) the Prodigal (PROkaryotic Dynamic Programming Gene-finding ALgorith) software (Hyatt et al., 2012) was used with parameterization for single genomes. The produced ORFs were then used as an input for BlastKoala (Kanehisa et al., 2016), which provides the Kyoto Encyclopedia of Genes and Genomes (KEGG) Orthology (KOs) assignments. The gene annotation was carried out using the databases, “species\_prokaryotes” or “genus\_prokaryotes” for the five *Oenococcus* strains. The KO and KEGG pathway enrichment analysis were done by custom python and R scripts (Melkonian et al., 2019); KEGGREST (Tenenbaum, 2016), lattice (Sarkar, 2008), apcluster (Frey and Dueck, 2007; Bodenhofer et al., 2011), Python BioServices (Cokelaer et al., 2013) and pandas (McKinney, 2011). In total, for all five *O. oeni* strains we obtain 857 unique KOs, which mapped to 151 unique KEGG metabolic pathways.

To identify potential genes coding for esterase activity, a local BLAST database was created with nine esterase genes reported in literature (Table 2). Therefore, each *O. oeni* strains ORFs were compared to the database and filtered with *e*-value < 0.05 and identity greater than 50%. For all significant hits of each strain, a multiple sequence alignment was performed together with the nine esterase genes using ClustalW algorithm with default parametrization. The resulting alignments were used to produce pairwise distance matrices and unrooted phylogenetic trees employing the msa (Bodenhofer et al., 2015), seqinr (Charif and Lobry, 2007), phytools (Revell, 2012), and ape (Paradis and Schliep, 2018) R-packages.

**TABLE 2:** Putative esterases from the literature.

**Microbial species studied**	**Esterase type**	**Enzyme name**	**Molecular weight (kDa)**	**Substrates**	**Gene involved**	**Gene sequence link**	**Article**
*Talaromyces stipitatus*	Feruloyl esterase	FAEC	55.3	Methyl ferulate, methyl caffeate, methyl sinapate, methyl *p*-coumarate	*faeC*	https://www.ncbi.nlm.nih.gov/nuccore/AJ505939	Crepin et al., 2003
*Lactobacillus johnsonii* NCC 533	Ferulic acid esterases	Lj1228 and Lj0536	31	Ethyl ferulate, chlorogenic acid	ND	https://www.ncbi.nlm.nih. gov/nuccore/GU454587.1 https://www.ncbi.nlm.nih. gov/nuccore/GU454586.1	Lai et al., 2009
*Lactobacillus jonhsonii* DPC6026	Cinnamoyl esterase	LJP-0936	ND	ND	ND	https://www.ncbi.nlm. nih.gov/protein/329667314	Guinane et al., 2011
*Lactobacillus plantarum* WCFS1	Feruloyl esterase/carboxyl esterase	Lp_0796	28	Methyl ferulate, methyl caffeate, methyl *p*-coumarate, and methyl sinapinate	*lp_0796*	https://www.ncbi.nlm. nih.gov/protein/YP_004888771.1	Esteban-Torres et al., 2013
7 *Lactobacillus plantarum*	Feruloyl esterase	Est-1092	33.5	Methyl ferulate, methyl caffeate	*est-1092*	GenBank accession number CP001617.1.	Esteban-Torres et al., 2015
*Oenococcus oeni* PSU-1	Esterase	EstB28	34.5	Volatile aromatic esters	*estB28*	https://www.ncbi.nlm.nih.gov/nuccore/410695969	Sumby et al., 2009
*Oenococcus oeni* and *Lactobacillus hilgardii*	Esterases	EstCOo8 and EstC34	29	Volatile aromatic esters	*estCOo8* and *estC34*	https://www.ncbi.nlm.nih.gov/nuccore/JX215240.1 https://www.ncbi.nlm.nih.gov/nuccore/418206119	Sumby et al., 2013

*ND, no data.*

A similar process was followed to identify possible transporter genes – each *O. oeni* strain ORFs was blasted against the local version of TCDB 2.0 database (Saier et al., 2006), filtered with *e*-value < 0.05 and identity bigger that 70%.

All *O. oeni* strains had a highly significant hit with EstB28 gene (WP_011677767.1, alpha/beta hydrolase) from *Oenococcus oeni* PSU-1 strain. Therefore, the corresponding upstream sequences transporter gene (WP_002823494.1, MFS transporter) and regulator gene (WP_002821683.1, AraC family transcriptional regulator) were also similarly searched in all five *O. oeni* strains.

### Chemical Analyses

#### Determination of the Concentrations in HCAs and Tartrate Esters

The analysis was performed following the method described in Oliveira et al. (2015), although with some modifications. The identification and quantification of phenolic compounds was conducted by an HPLC system with diode array detection (DAD) from Waters Corporation (Milford, MA, United States). The stationary phase used was a Kromasil^®^ C18 HPLC column 5 μm × 250 mm × 4.6 mm from Sigma-Aldrich (Steinheim, Germany). A binary HPLC solvent system was used with the following mobile phases: phase A, composed of acetonitrile from Fisher Chemical (Pittsburgh, PA, United States) with 0.2% of trifluoroacetic acid (TFA) (Sigma-Aldrich, Germany) and phase B, a mixture of acetonitrile and ultra-pure water 5: 95 v/v with 0.2% TFA with the following gradient: 0–1 min (100% B); 1–30 min (100% to 79% B); 30–42 min (79% to 73% B); 42–55 min (3% to 42% B), 55–60 min (42-100% B), and 60–61 min (100% B). The absorption spectra of all peaks were recorded between 212 and 600 nm. HCAs and tartrate esters were detected at 320 nm and identified according to their UV-vis spectra and retention times of known standards. Standards of *trans*-caftaric, *trans*-*p*-coumaric, *trans*-ferulic and *trans*-caffeic acids (purities ≥ 98%) were obtained from Sigma-Aldrich (Steinheim, Germany). The suitability of the ferulic acid methyl ester - that has been used in other studies of this type (Crepin et al., 2003; Esteban-Torres et al., 2013, 2015) was tested too as substrate in the esterase activity experiment. This compound (purity ≥ 95%) was obtained from Extrasynthese (Genay, France). The concentrations were calculated according to standard calibration curves. The tartrate esters with unavailable standards were quantified as *trans*-caftaric acid equivalents using the corresponding calibration curves. Samples were filtered (0.45 μm) and directly injected without dilution.

#### Protein Quantification

The protein contents of the cell extracts and cell debris of the five *O. oeni* strains from CH. Hansen cultivated in unexposed and wine-exposed media were evaluated by the Bradford method (Martina and Vojtech, 2015). To avoid interference with lysozyme, only the cell extracts and cell debris of strains lysed with the mechanical protocol were used for the analysis of protein concentration. Protein quantification was performed in triplicate for each sample at once using the same calibration, and the results were given in mg/L for 1 mL suspension and 1 AU of culture.

### Statistical Analysis

All experiments were performed with a minimum of two replicates. Data were subjected to statistical analysis using JMP 13 for Windows XP (JMP, Marlow, United Kingdom), at a confidence level of 95% (*p* = 0.05). A Tukey’s–Kramer HSD (honestly significant differences) test was run to compare yield values between samples of different sizes. A one-way analysis of variance (ANOVA) was used to determine the minimum significant difference (*p* < 0.05) between media for the protein results.

## Results

### Screening for Cinnamoyl Esterase Activity

Of the 15 strains tested, only *O. oeni* Oenos^TM^, CH35^TM^ and CiNe^TM^ showed cinnamoyl esterase activity (CE+), and these were expressed at different levels, depending on the strain. The degradation of *trans*-caftaric, *trans*-coutaric and *trans*-fertaric acids by Oenos^TM^ and CiNe^TM^ was almost complete (≥88%) whilst only 30% was recorded for CH35^TM^.

The molar conversion of *trans*-coutaric and *trans*-fertaric acids into their HCA free forms was less complete than the molar conversion recorded for *trans*-caftaric acid (Table 3).

**TABLE 3:** Molar percentages (%n) of free HCAs released from their tartrate derivatives precursors by the three CE+ *O. oeni* strains.

	**Oenos^TM^**	**CiNe^TM^**	**CH35^TM^**
*Trans*-caffeic/*trans*-caftaric acids	87	89	77
*Trans*-*p*-coumaric/*trans*-coutaric acids	60	65	65
*Trans*-ferulic/*trans*-fertaric acids	40	40	34

The concentration in *cis-*caftaric and *cis*-coutaric acids remained unchanged contrary to their *trans*-isomer forms (Supplementary Figure S1).

### Evaluation of *O. oeni* Growth Inhibition by *Trans*-Caftaric Acid, *Trans*-Caffeic Acid and 4-ethylcatechol

**FIGURE 2:**
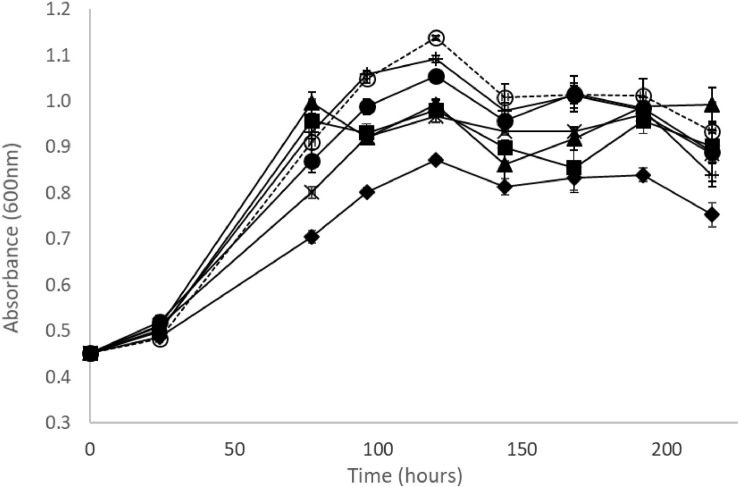
Growth curves of *O. oeni* Oenos^TM^ in MRS broth medium (pH 4.5, 5% v/v ethanol at 25°C with no agitation in aerobic conditions) supplemented with (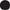) 150 mg/L and (+) 300 mg/L of *trans*-caftaric acid, (x) 85 mg/L and (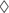) 170 mg/L of *trans*-caffeic acid, (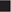) 65 mg/L and (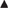) 130 mg/L of 4-ethylcatechol, (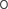) no phenolics added; error bars represent the standard deviation of three determinations.

Within the range of concentrations tested, the tartrate ester of *trans*-caffeic acid (*trans*-caftaric acid) apparently did not inhibit the growth of Oenos^TM^, whilst *trans*-caffeic itself and its corresponding ethyl-phenol (4-ethylcatechol), added at the same molar concentrations (0.5 and 1 mmol/L) in culture medium did inhibit, with a stronger effect at the higher concentrations tested (Figure 2). Similar observations were made for the four other *O. oeni* strains under study (Supplementary Figure S2).

### Gene Comparison Between *O. oeni* Genomes

**FIGURE 3:**
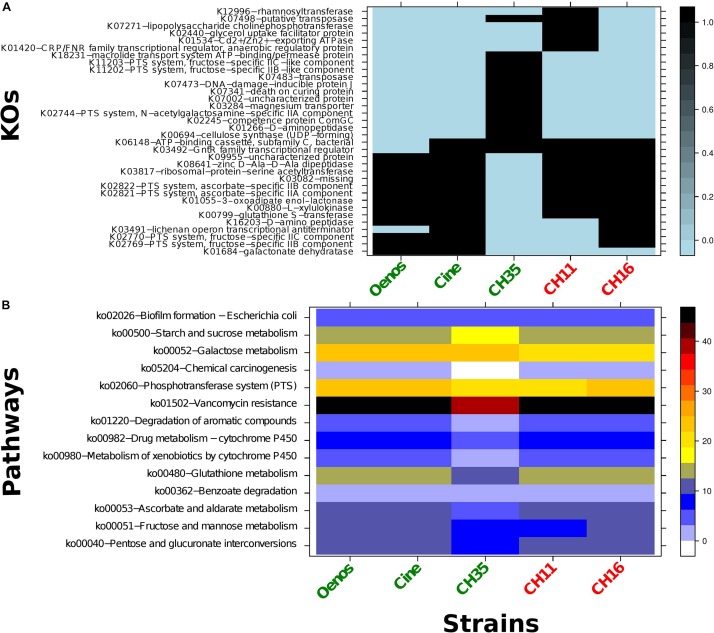
**(A)** Heatmap representing the distribution of the 34 different KOs within the five *O. oeni* strains. Black - presence of the KO; light blue - absence of the KO; green - CE + strains and red - CE- strains. Row-names correspond to the KO identifiers and the corresponding KO definition. **(B)** Heatmap of the KEGG pathways enrichment within the five *O. oeni* strains. In right side the color scheme represents the pathway coverage which is the percentage of the KOs mapping the pathway divided by the total KOs number for the corresponding pathway.

The KO annotations of the five *O. oeni* strains were compared with each other and from the 857 KOs 34 were found to be different between the strains. As observed in Figure 3, no known KO esterase nor other gene that could explain the presence of absence of cinnamoyl esterase activity was exclusive to the three CE+ strains.

As has been previously applied, seven published ORFs encoding CE in the *Lactobacillus* genus and two others coding esterases in wine *Oenococcus* genus were selected (Table 2) and computationally analyzed against the ORFs of the five targeted *O. oeni* strains (Supplementary Figure S3 – example for Oenos^TM^). A good hit (99.01% - 99.34% identity with 0 *e*-value) for all the strains toward the EstB28 esterase of *O. oeni* PSU-1 was observed (Sumby et al., 2009). On four strains (all except CH35^TM^) two fragmented ORFs were blasted both with good hits as well (95.91 and 96.34% identities with 4e-123 and 1e-54 *e*-values) toward the EstCOo8 esterase from Sumby et al. (2013) study. Other ORFs could also be pointed out with relatively good hits (above 70% identity and *e*-value < 0.05) against FAEC feruloyl esterase from *Talaromyces stipitatus* (Crepin et al., 2003) and Lj0536 ferulic acid esterase from *Lactobacillus johnsonii* NCC 53 (Lai et al., 2009).

A synteny analysis on EstB28 gene and its corresponding regulator and transporter genes was performed to determine if differences between the CE+ and CE− strains could be detected upstream. Once more, significant hits (above 99% identity and *e*-value 0) toward all *O. oeni* strains were found for transporter gene (WP_002823494.1, MFS transporter) and regulator gene (WP_002821683.1, AraC family transcriptional regulator).

A search of all transporters genes with the usage of TCDB 2.0 database was performed for the five *O. oeni* strains. In total twenty-two ORFs had a significant hit with fifteen commonly shared between the strains (Supplementary Figure S4).

### Cellular Effect of Prior Exposure to Wine and Localization of Enzymatic Activity

This experiment aimed to explore the potential enzyme induction effect of prior exposure of the strains to wine – as a source of multiple, potentially inducing substrates.

#### Effect of 30% v/v Wine on Growth of *O. oeni* Strains

Prior exposure to wine (30% pasteurized wine) slightly activated the bacterial growth of CiNe^TM^, CH35^TM^ and CH16^TM^ although the level of enhancement was strain dependent (CiNe^TM^ and CH35^TM^, 8.83 log CFU mL^–1^ for unexposed strains after 8 days incubation against 9.32 log CFU mL^–1^ for wine-exposed strains, and CH16^TM^ 9.01 log CFU mL^–1^ for unexposed strains after 5 days incubation against 10.02 log CFU mL^–1^ for wine-exposed strains).

#### Cellular Localization of Enzymatic Activity

Comparing the efficiency of the two lysis protocols used, 40% of the *O. oeni* cells were disrupted by the mechanical method against 60% by the enzymatic one. No activity was detected in the extracellular parts (supernatants) of the non-lysed CE+ strains.

All cell-free extracts of the *O. oeni* strains tested in wine-exposed (30% pasteurized wine) and unexposed media, except CH11^TM^, appeared to show a cinnamoyl esterase activity. However, the activity was stronger in the cell-free extracts of the CE+ strains. An almost immediate reaction to exposure to the cell extracts, leading to the total degradation of *trans*-caftaric acid, was observed for extracts from the three CE+. More than 80% of the *trans*-caftaric acid consumed during this period apparently liberated *trans*-caffeic acid. As for CH16^TM^, a partial activity in the cell-free extracts was found, and this was higher in the experiments where there was no prior exposition to the wine (Table 4). Although the cell lysis efficiency was greater with the enzymatical protocol compared to the mechanical one, for the wine-exposed CH16^TM^ strains, the cinnamoyl esterase activity was lower.

**TABLE 4:** Cinnamoyl esterase activity of cell-free extracts of *O. oeni* strain CH16^TM^, grown in MRS medium mixed (wine-exposed) or not (unexposed) with 30% pasteurized wine, obtained after applying the mechanical and enzymatic lysis protocols.

		**log (CFU/mL) before cell lysis**	***Trans*-caftaric acid (mg/L)**		***Trans*-caffeic acid (mg/L)**		**% *trans*-caftaric degraded**
					
			**t0**	**tfinal**	**t0**	**tfinal**	
Mechanical lysis	Unexposed	8.81 ± 0.12	9.91 ± 0.82	2.44 ± 2.21	0.51 ± 0.35	4.04 ± 0.96	76 ± 22
	Wine-exposed	9.50 ± 0.11	11.21 ± 0.40	8.80 ± 0.60	0.19 ± 0.06	1.14 ± 0.15	22 ± 4
Enzymatic lysis	Unexposed	8.71 ± 0.05	10.77 ± 0.46	3.42 ± 2.49	0.14 ± 0.03	3.21 ± 1.31	68 ± 25
	Wine-exposed	9.44 ± 0.10	11.55 ± 0.25	10.88 ± 0.01	0.11 ± 0.00	0.44 ± 0.13	6 ± 2

*Values represent the mean of four values ± standard deviation. t0 – immediately after substrate addition and before incubation. tfinal – after incubation at 30°C for 16 h.*

The protein concentration in the cell extracts of Oenos^TM^, CH16^TM^ and CH11^TM^ strains grown in unexposed medium (pure MRS broth) was two to three times higher than after exposition to 30% pasteurized (Supplementary Table S1). Only in the case of the CE+ strains exposed to wine did the cell debris contain higher protein concentrations that the unexposed ones.

Ferulic acid methyl ester was observed not to be substrate for the cinnamoyl esterase activity of the 5 *O. oeni* strains studied, neither in the extracellular medium, nor in the cell-free extracts.

### Cinnamoyl Esterase Activity in Live Cultures; Strain Differences and Prior Exposure Effects

**FIGURE 4:**
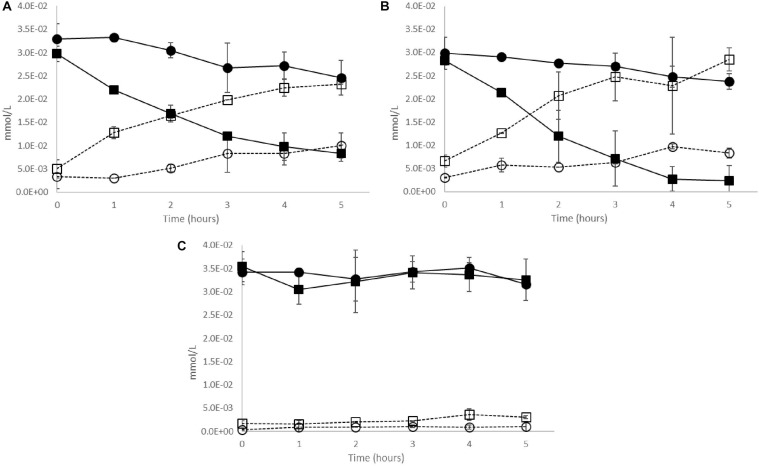
Cinnamoyl esterase activity in live cultures of *O. oeni* CE+ strains **(A)** Oenos^TM^, **(B)** CiNe^TM^, and **(C)** CH35^TM^ previously grown in (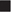, 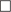) wine-exposed or (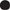, 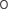) unexposed media. Solid lines – *trans*-caftaric acid degradation; dotted lines – *trans*-caffeic acid production. Error bars represent the standard deviation of three determinations.

In the case of the two CE+ strains Oenos^TM^ and CiNe^TM^, *trans*-caftaric acid was degraded faster by the cells previously grown in 30% pasteurized wine (wine-exposed) comparatively to the cells grown without prior exposition to the wine (unexposed) (Figures 4A,B). The reaction was slightly faster for the wine-exposed cells of CiNe^TM^ (Figure 4B). Almost no degradation was registered for CH35^TM^ over 5 h of incubation (Figure 4C).

In order to evaluate the stimulation of the cinnamoyl esterase activity, cultures of the CE+ strain Oenos^TM^ were grown without agitation for 5 days and 10 h at 25°C in different media with a certain time of substrate-exposure (5 days and 10 h or only 10 h). All *trans*-caftaric acid was apparently degraded by the live culture of *O. oeni* Oenos^TM^ after 5 days and 10 h growth and exposure at 25°C (Table 5).

**TABLE 5:** *Trans*-caftaric and *trans*-caffeic acids concentrations in different cultures of *O. oeni* Oenos^TM^ before (t0) and after (tfinal) 5 days and 10 h growth and a certain time of substrate-exposure (5 days and 10 h or only 10 h) at 25°C.

**Medium culture**	****log (CFU mL^–1^) tfinal****	***Trans*-caftaric acid (mg/L)**		***Trans*-caffeic acid (mg/L)**	
			
		**t0**	**tfinal**	**t0**	**tfinal**
(1) Unexposed	8.87 ± 0.07	*nd*	*nd*	*nd*	*nd*
(2) 10% wine-exposed for 5 days and 10 h	9.30 ± 0.07	16.59 ± 0.11	*nd*	0.19 ± 0.00	8.80 ± 0.14
(3) 20% wine-exposed for 5 days and 10 h	9.73 ± 0.36	33.97 ± 0.13	*nd*	0.28 ± 0.01	18.13 ± 0.02
(4) 30% wine-exposed for 5 days and 10 h	9.68 ± 0.13	50.74 ± 0.58	*nd*	0.39 ± 0.02	26.85 ± 0.08
(5) 50 mg/L *trans*-caftaric acid for 5 days and 10 h	8.87 ± 0.10	49.45 ± 1.56	*nd*	*nd*	19.97 ± 0.86
(6) 20% wine-exposition for the last 10 h incubation	9.45 ± 0.08	33.97 ± 0.13	20.42 ± 0.00	0.28 ± 0.01	5.68 ± 0.00
(7) 50 mg/L *trans*-caftaric acid for the last 10 h incubation	8.90 ± 0.09	49.45 ± 1.56	29.73 ± 0.05	*nd*	8.27 ± 0.02

*nd, not detected (detection limit of *trans*-caftaric and *trans*-caffeic acids = 0.10 mg/L). Values represent the mean of two to three values ± standard deviation.*

Regardless the proportion of pasteurized wine added to the culture medium and the time of exposure (5 days and 10 h or only 10 h), the rate of the cinnamoyl esterase activity of *O. oeni* Oenos^TM^ was relatively similar and, in all cases, higher than with added pure *trans*-caftaric acid as a potential stimulator and without any prior addition of wine (Table 6).

**TABLE 6:** Cinnamoyl esterase activity in cultures of *O. oeni* Oenos^TM^ previously grown in different culture media, in KH_2_PO_4_ buffer (0.15 M, pH 4.5, 9% EtOH) at 25°C supplemented with *trans*-caftaric acid.

**Medium culture**	***Trans*-caftaric acid (mg/L)**		***Trans*-caffeic acid (mg/L)**		**% *trans-*caftaric acid degraded**
			
	**t0**	**t13**	**t0**	**t13**	
(1) Unexposed	13.72 ± 0.65	9.49 ± 0.62	0.25 ± 0.08	2.13 ± 0.25	31 ± 1^*b*^
(2) 10% wine-exposed for 5 days and 10 h	9.33 ± 0.18	2.58 ± 0.15	2.45 ± 0.12	4.81 ± 0.15	72 ± 1^*a*^
(3) 20% wine-exposed for 5 days and 10 h	12.65 ± 0.60	3.92 ± 1.38	1.50 ± 0.25	4.84 ± 0.54	69 ± 12^*a*^
(4) 30% wine-exposed for 5 days and 10 h	11.82 ± 0.30	3.54 ± 0.58	1.67 ± 0.10	5.08 ± 0.13	70 ± 4^*a*^
(5) 50 mg/L *trans*-caftaric acid for 5 days and 10 h	11.20 ± 1.36	7.92 ± 0.12	1.18 ± 0.08	3.24 ± 0.48	29 ± 8^*b*^
(6) 20% wine-exposition for the last 10 h incubation	12.97 ± 0.00	2.30 ± 0.00	1.84 ± 0.00	6.28 ± 0.00	82 ± 0^*a*^
(7) 50 mg/L *trans*-caftaric acid for the last 10 h incubation	14.89 ± 0.35	9.94 ± 0.94	0.33 ± 0.00	2.65 ± 0.01	33 ± 5^*b*^

*Values represent the mean of two to three values ± standard deviation. Values followed by different letters within a column are statistically significantly different at *p* < 0.05 using a Tukey’s–Kramer HSD test. t0 – immediately after *trans*-caftaric acid addition and before incubation. t13 – after incubation at 25°C for 13 h.*

## Discussion

Wine spoilage by the production of volatile phenols is thought to be influenced by the liberation of precursor HCAs from their tartrate ester forms by the cinnamoyl esterase activity of a few specific LAB strains (Chescheir et al., 2015; Madsen et al., 2016). However, the characteristics of the enzymatic activity have not been explored. Various microbial CE have been studied, purified and characterized but none of these had tartrate esters as recognized substrates (Sumby et al., 2013; Esteban-Torres et al., 2015; Song and Baik, 2017). Thus, this paper brings new insights into the enzymatic activity in *O. oeni*.

Viniflora^®^ Oenos^TM^, CH35^TM^, Enoferm Alpha^TM^ and Lalvin VP41^TM^ formerly tested by Chescheir et al. (2015) for their cinnamoyl esterase activity in wine, Viniflora^®^ CiNe and CH11^TM^ described respectively as CE+ and CE− in Madsen et al. (2016) study, the LAB reported in Couto et al. (2006) work associated in some way with the production of volatile phenols, together with the commercial *O. oeni* strain Viniflora^®^ CH16^TM^ and *L. plantarum* strain NOVA^TM^ were screened in the current study for their cinnamoyl esterase activity in a medium culture using 30% of pasteurized wine as a source of tartrate esters. The three *O. oeni* strains Oenos^TM^, CH35^TM^ and CiNe^TM^ were able to release free HCAs from their tartrate ester forms at different yields, with *O. oeni* CH35^TM^ having the lowest conversion yield, suggesting that the esterase system might somehow differ between strains. Despite the degradation of *trans*-coutaric and *trans*-fertaric acids by CE, as registered by their disappearance from the samples analyzed, their free HCA forms (*trans-p-*coumaric and *trans-*ferulic acids) did not yield their molar equivalent levels. It is possible that, as these compounds are relatively reactive, the HCAs liberated might be converted to other reaction products (Singleton et al., 1985). Another hypothesis would be that the cinnamoyl esterase studied could have more affinity toward *trans*-caftaric acid than to other substrates. In accordance with Hernandez et al. (2007), the *trans*-forms such as *trans-*caftaric and *trans-*coutaric acid were the only ones cleaved in this process.

The five commercial *O. oeni* from Ch. Hansen were further tested in this study; Oenos^TM^, CH35^TM^ and CiNe^TM^ exhibiting cinnamoyl esterase activity (CE+) and CH11^TM^ and CH16^TM^ not exhibiting it (CE−).

Computation analysis on the genomic information of the five *O. oeni* strains was carried out in an attempt to identify the genetic basis of the observed differences between CE+ and CE− phenotypes. No specific genes common only to the three CE+ strains could explain the different activity. This observation suggests the potential involvement of more than a single enzyme in the cinnamoyl esterase activity of *O. oeni* strains tested. A good hit was observed in all strains toward a single, known esterase gene related to aroma esters (EstB28). The presence of the corresponding regulator and transporter genes were explored and identified, but the results were in-conclusive. Another interpretation could be that genes that may be relevant for the metabolism (or transport) of the targeted substrates were not expressed in the case of the CE− strains.

The effects of HCAs on the growth and metabolism of LAB have been widely reported, both in scenarios related to wine production and others. The decrease in cell culture viability of Oenos^TM^ by free HCAs has been linked to the increase of the cell membrane permeability by Campos et al. (2003, 2009). At the maximum concentration used (1 mmol/L), unlike its metabolic products *trans-*caffeic acid (from the cinnamoyl esterase metabolism) and 4EC (from the volatile phenols metabolic pathway), *trans-*caftaric acid had no effect on the growth of the five *O. oeni* strains in MRS medium broth. Since Oenos^TM^, CiNe^TM^ and CH35^TM^ are cinnamoyl esterase positive strains, a stronger inhibitory effect of *trans-*caftaric acid than of *trans*-caffeic acid would be expected assuming that detoxification was the main biological mechanism involved. Therefore, the presence of cinnamoyl esterase activity doesn’t seem to be justifiable by a stronger inhibitory effect from *trans*-caftaric acid compared to *trans-*caffeic acid and 4EC.

In the current study, the addition of 30% of pasteurized wine as source of tartrate esters slightly increased the bacterial growth of CiNe^TM^, CH35^TM^ and CH16^TM^. This observation may be partially explained by the presence of wine components which may have stimulated bacterial growth.

Several cell disruption buffers and chemical, mechanical or enzymatic techniques are regularly employed in the lab to liberate the intracellular content of LAB for a number of purposes (Couto and Hogg, 1994; Lai et al., 2009; Ya-hui et al., 2012; Esteban-Torres et al., 2013, 2015; Cafaro et al., 2014). Among the 13 different lysis protocols tested, only two were able to adequately disrupt the cell wall and membrane of the five commercial *O. oeni* strains studied; one enzymatic and one mechanical protocol. The esterase enzyme targeted in this study was found in the cell-free extracts of the strains, as those previously studied by Sumby et al. (2009, 2013).

Sommer et al. (2018) suggest that lysozyme addition to wine prior to fermentation may affect the release of HCAs from their tartrate derivative forms. In this present study, in the control without bacteria, no *trans*-caftaric acid cleavage was observed even where laboratory grade lysozyme was employed in the lysis protocol.

The cinnamoyl esterase activity was found in both wine-exposed and unexposed cell extracts of the CE+ strains. The significance of this is still unclear but one possibility might be that the enzyme(s) responsible have another, perhaps more generic, role in the biology of the bacteria that show this activity.

Interestingly, the ferulic acid methyl ester used as a substrate in other related studies (Crepin et al., 2003; Esteban-Torres et al., 2015) was observed not to be substrate for the activities shown by the strains tested in this study, neither in the extracellular medium, nor in the cell extracts. This phenomenon could possibly be due to the short side chain of this compound comparatively to the tartrate esters or to tartaric acid (or any acid) moiety which might be necessary for the enzyme activity.

Among the CE+ strains tested, CH35^TM^ was found to have the lowest cinnamoyl esterase activity. However, after lysis and cell contents release, the substrate was almost instantly cleaved. Moreover, CH16^TM^, considered to be CE−, partially degraded *trans*-caftaric acid once the cell contents were liberated. These results suggest a possible role for the wall and/or membrane in the activity studied. The pK_A_ values of free forms of phenolic acids ranges from 4.2 to 4.5 (Ramos-Nino et al., 1996). At the pH of the media used in these experiments (4.3–4.5) about half of the total phenolic acids’ concentration would be in the un-dissociated forms and thus is expected to cross the cell membrane by passive diffusion (Campos et al., 2009). There is a possibility that tartrate esters bind or are blocked by cell wall components and therefore could not cross the membrane, or that these compounds require some form of active or facilitated membrane transport in order to enter the cells. However, analysis of the Venn diagram of transporter genes from the five *O. oeni* strains did not suggest a common gene present only in the 3 CE+ strains.

A higher activity was noted for the CH16^TM^ cell extracts with no prior exposition to the wine which suggests that some wine components might interfere with the ability of *O. oeni* CH16^TM^ to degrade *trans*-caftaric acid.

Cinnamoyl esterase activity was faster in the CE+ live cultures that have been previously exposed to wine which could possibly be due to membrane protein transporters expressed on prior wine exposure. The protein content was higher in the cell debris of the CE+ strains exposed to wine, which could be explained by the stress application caused by wine addition, as observed in a previous study (Garbay and Lonvaud-Funel, 1996). When added to give the same content in *trans*-caftaric acid (50 mg/L), pasteurized wine (30%) was apparently better at stimulating the esterase activity than the pure molecule alone. This observation suggests that other wine compounds may be involved in the stimulation of this enzymatic activity. Previously, other authors have suggested that free HCAs may induce PAD and VPR activities in wine LAB (Silva et al., 2011) and that the concentration of *p-*coumaric acid is the most significant factor correlated with the expression of the gene coding for PAD in *L. plantarum* isolates (Rosimin and Kim, 2015). Free HCAs could also have induced the synthesis of enzymes involved in the metabolism of *trans-*caftaric acid.

Altogether, the results presented suggest the possibility of the involvement of more than merely a single catalytic enzyme in the production of free HCAs from their tartrate derivative forms in wine by *O. oeni*, a potential stimulation of the activity by wine related molecules and a cell-free extracts location of the esterase molecule itself. Further studies will be needed to characterize the enzymatic activity.

Characterization of esterase activity by wine components, purification of the intracellular esterase by non-destructive methods such as FPLC (Fast protein liquid chromatography) technique and gene expression analysis by transcriptomics would be appropriate approaches to validate this suggestion (Rossouw et al., 2012; Sumby et al., 2013; Wang et al., 2016; Hwang et al., 2018).

## Data Availability Statement

The datasets generated for this study can be found in the GenBank (CiNeTM) AZJV00000000, GenBank (CH35TM) ALAG0000000, and 3 others provided and approved by the company Ch. Hansen can be found in https://doi.org/10.5281/zenodo.3515872 (OenosTM, CH11TM and CH16TM).

## Author Contributions

IC and TH conceived the idea. IC designed the experiments, carried out the laboratory work, performed the analyses and interpretations of the experimental data and wrote the manuscript, except the bioinformatics part, with inputs from all authors. CM led the analysis and interpretation of bioinformatics data and wrote the bioinformatics part of the manuscript. TH, FC, and DM oversaw the study and edited the manuscript. All authors read and approved the final version of the manuscript.

## Conflict of Interest

The five *O. oeni* strains and their genomes were provided by Ch. Hansen. The authors declare that the research was conducted in the absence of any commercial or financial relationships that could be construed as a potential conflict of interest.
