# Supplementary material for: New Insights Into Cinnamoyl Esterase Activity of Oenococcus oeni
Source: Front Microbiol. 2019 Nov 8;10:2597. doi: 10.3389/fmicb.2019.02597 (PMC6857119; doi:10.3389/fmicb.2019.02597)
Supplement: Supplementary file 1 [file Table_1.DOCX]

Supplementary Material

**
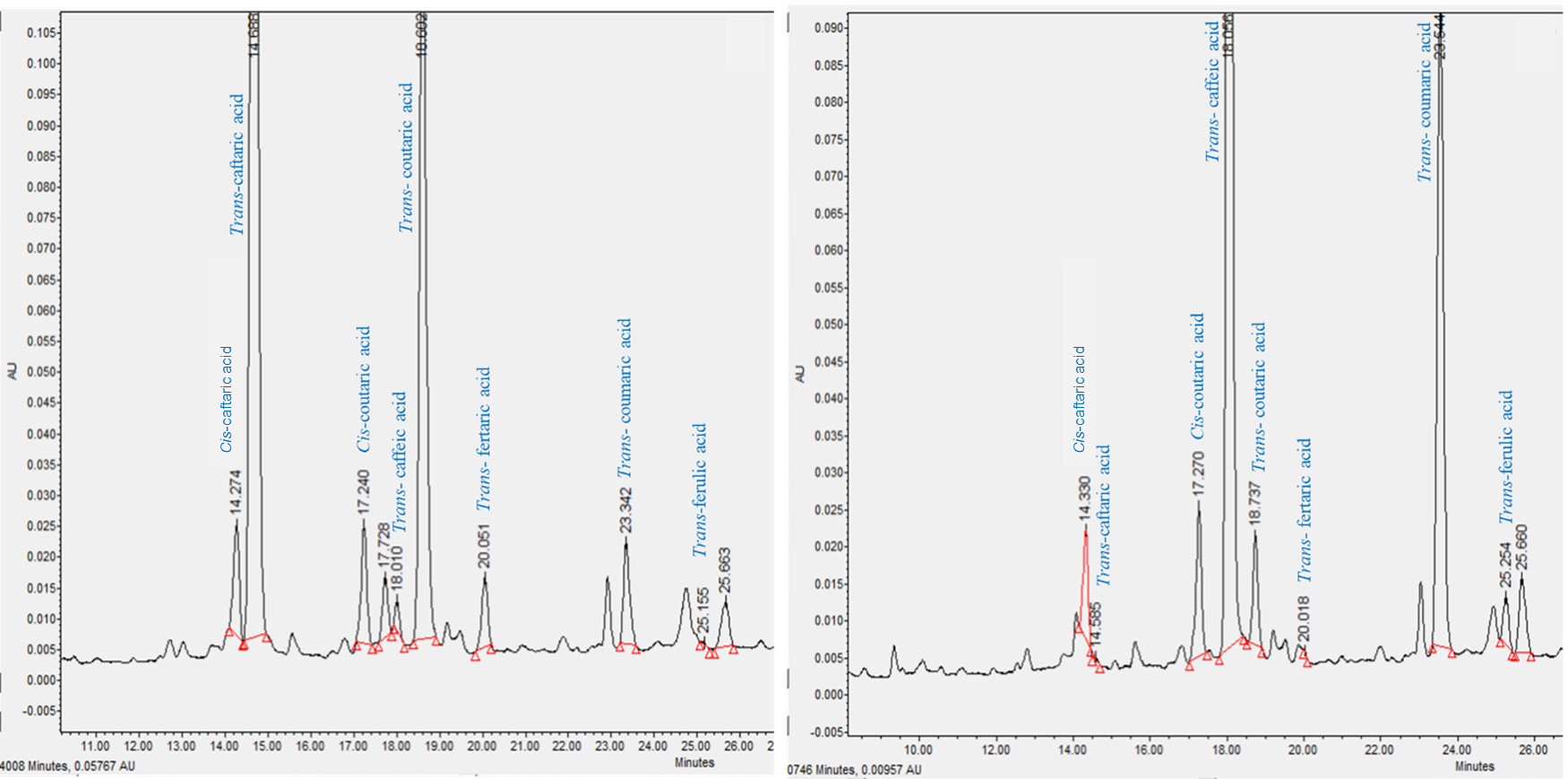
**

**(B)**

**(A)**

**Supplementary Figure S1.** HPLC-DAD chromatograms (at 320 nm wavelength) of the HCAs (*trans*-caffeic, *trans*-coumaric and *trans*-ferulic acids) and corresponding tartrate esters (*trans*-caftaric, *cis*-coutaric, *trans*-coutaric and *trans*-fertaric acids) in a *O. oeni* Oenos^TM^ culture grown in 30 % pasteurized wine and 70% MRS broth; (A) before and (B) after incubation at 25 °C with no agitation until log (CFU mL^-1^) ~ 9.26 (± 0.21).

**
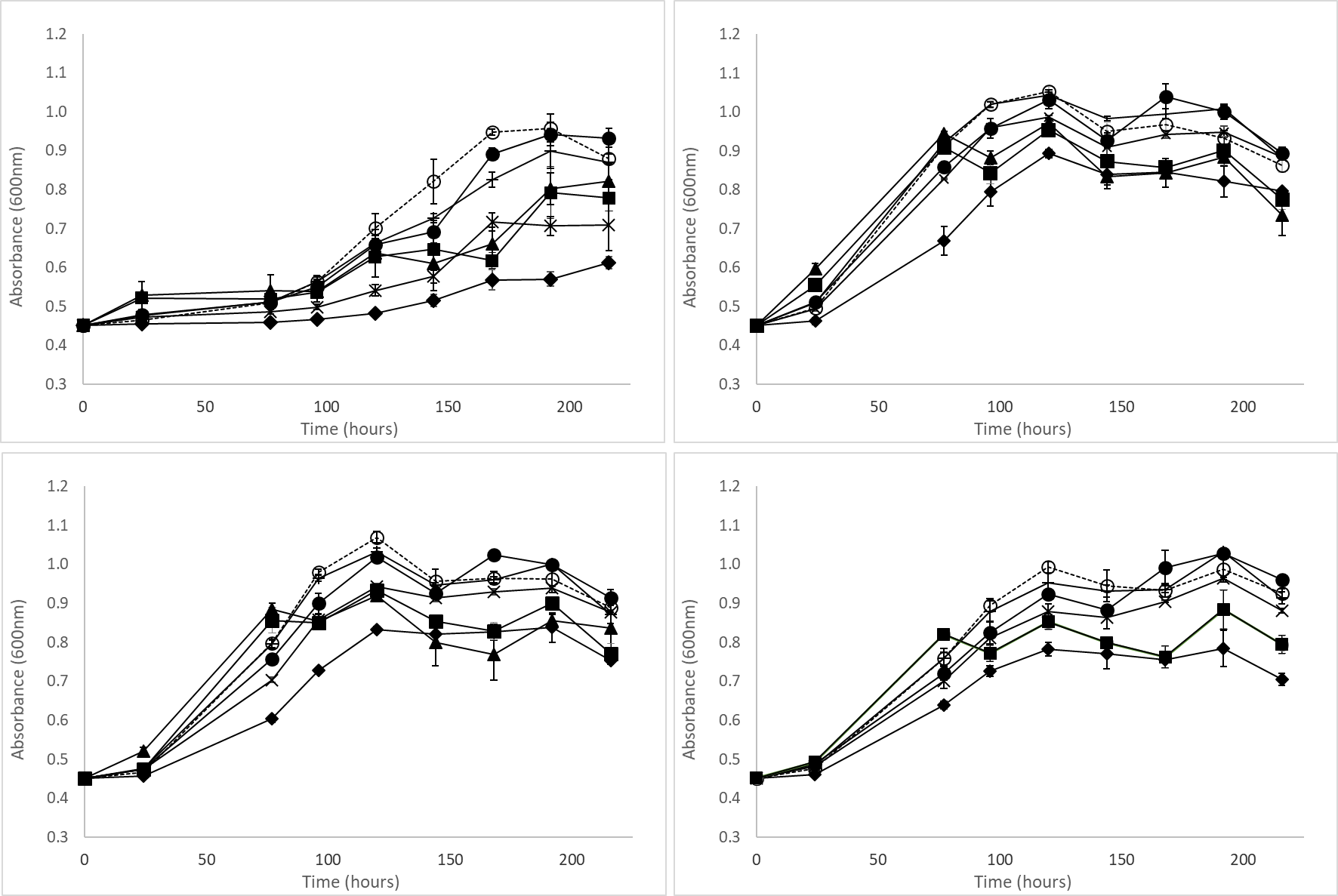
**

**(D)**

**(C)**

**(B)**

**(A)**

**Supplementary Figure S2.** Growth curves of *Oenococcus oeni* (A) CiNe^TM^, (B) CH35^TM^, (C) CH16^TM^ and (D) CH11^TM^ in MRS broth medium (pH 4.5, 5% v/v ethanol at 25 °C with no agitation in aerobic conditions) supplemented with (●) 150 mg/L and (+) 300 mg/L of *trans*-caftaric acid, (x) 85 mg/L and (♦) 170 mg/L of *trans*-caffeic acid, (■) 65 mg/L and (▲) 130 mg/L of 4-ethylcatechol, (○) no phenolics added; error bars represent the standard deviation of three determinations.

**
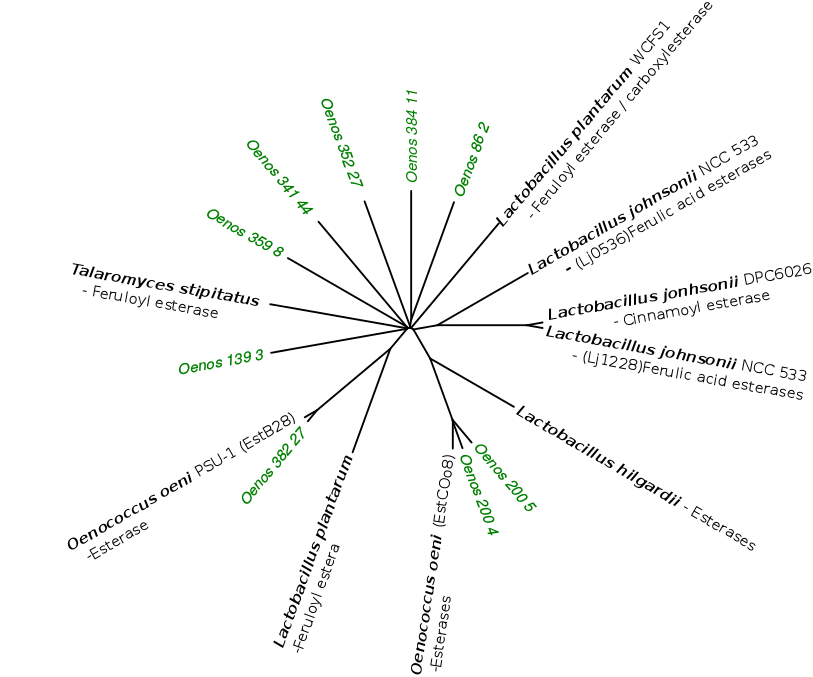
**

**Supplementary Figure S3.** Unrooted phylogenetic tree of all significant hits of Oenos^TM^ ORFs together with the nine esterase genes from the literature ([Crepin et al., 2003](#_ENREF_1); [Lai et al., 2009](#_ENREF_5); [Sumby et al., 2009](#_ENREF_7); [Guinane et al., 2011](#_ENREF_4); [Esteban-Torres et al., 2013](#_ENREF_3); [Sumby et al., 2013](#_ENREF_6)b; [Esteban-Torres et al., 2015](#_ENREF_2)).

**
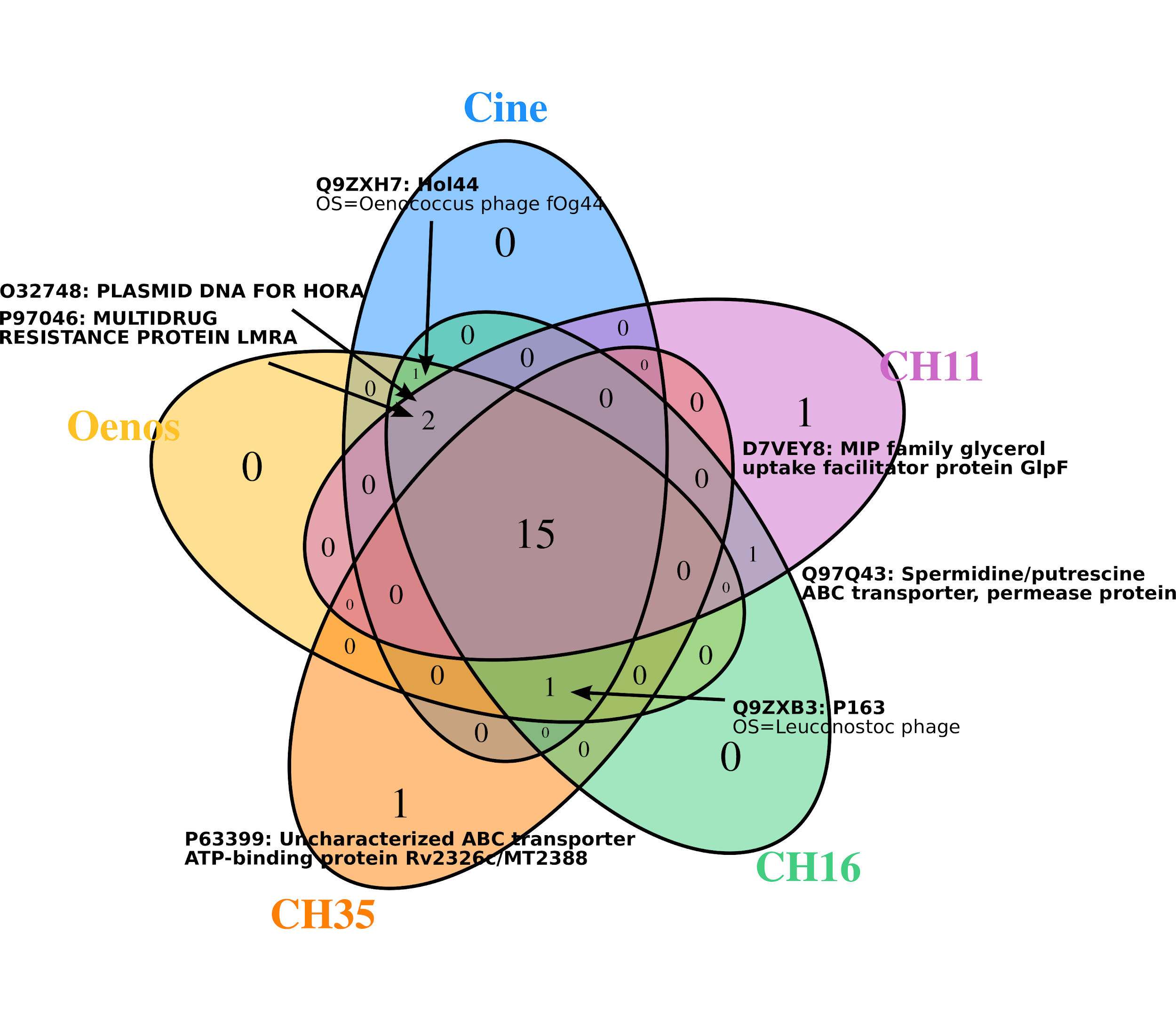
**

**Supplementary Figure S4.** Venn diagram of transporters genes from the five *O. oeni* strains based on custom orthology detection using TCDB 2.0 database.

**Supplementary Table S1.** Total protein content (mg/L) in 1 mL of cell-free extracts and cell debris of unexposed and wine-exposed *O. oeni* cultures. Values represent the mean of six values ± standard deviation.

|  | **Oenos^TM^** | | **CiNe^TM^** | | **CH35^TM^** | | **CH16^TM^** | | **CH11^TM^** | |
| --- | --- | --- | --- | --- | --- | --- | --- | --- | --- | --- |
|  | **Unexposed** | **Wine-exposed** | **Unexposed** | **Wine-exposed** | **Unexposed** | **Wine-exposed** | **Unexposed** | **Wine-exposed** | **Unexposed** | **Wine-exposed** |
| **Cell extracts** | 0.21 ± 0.01 | 0.08 ± 0.01 | 0.11 ± 0.01 | 0.09 ± 0.01 | 0.06 ± 0.01 | 0.05 ± 0.01 | 0.18 ± 0.01 | 0.06 ± 0.01 | 0.15 ± 0.01 | 0.09 ± 0.01 |
| **Cell debris** | 0.08 ± 0.02 **b** | 0.12 ± 0.01 **a** | 0.11 ± 0.02 **b** | 0.14 ± 0.00 **a** | 0.05 ± 0.00 **b** | 0.08 ± 0.01 **a** | 0.11 ± 0.01 | 0.10 ± 0.00 | 0.09 ± 0.01 | 0.09 ± 0.01 |

*Values followed by different bold letters are statistically significantly different at p-value < 0.001 using a One-way ANOVA.*
